# The Ebola epidemic: a transformative moment for global health

**DOI:** 10.2471/BLT.14.151068

**Published:** 2015-01-01

**Authors:** Stephen B Kennedy, Richard A Nisbett

**Affiliations:** a Liberia Post Graduate Medical Council, Corner of 12th Street and Russell Avenue, 2nd Floor Office Complex, Monrovia, 10001, Liberia.; b Vanderbilt Institute of Global Health, Nashville, United States of America.

The devastating effects of the current epidemic of Ebola virus disease in western Africa have put the global health response in acute focus. The index case is believed to have been a 2-year-old child in Guéckédou, Guinea, who died in December 2013.^1^ By late February 2014, Guinea, Liberia and Sierra Leone were in the midst of a full-blown and complex global health emergency.^2^ The response by multilateral and humanitarian organizations has been laudable and – at times – heroic. Much of the worst affected region is recovering from civil conflicts. This region is characterized by weak systems of government and health-care delivery, high rates of illiteracy, poverty and distrust of the government and extreme population mobility across porous, artificial boundaries. A more coordinated, strategic and proactive response is urgently needed.

According to the World Health Organization (WHO), the outbreak had involved 17 145 probable, suspected or confirmed cases of Ebola virus disease and 6070 reported deaths, by 3 December 2014.^3^ The management of the outbreak has largely been taken out of the hands of the affected communities, even though such communities have cultural mechanisms and expertise to deal with various adversities. Local churches and community-based organizations, which have previously been involved in the response to health emergencies and conflicts, have been largely excluded. Although the worst-affected communities have been subject to quarantines and *cordons sanitaires*, the governments imposing these have often failed to provide adequate food and water to the people thus isolated. In addition, *cordons sanitaires* are hard to maintain when local police and military personnel are not trusted.

Although it is difficult to build trust and community support during an Ebola outbreak, the community-directed interventions developed by the WHO’s Special Programme for Training and Research in Tropical Diseases^4^ might usefully be implemented. The interventions are designed to prevent, treat and control infectious diseases of poverty by empowering and mobilizing communities and building effective cross-sectoral partnerships. To be effective in addressing salient transborder health issues, global health initiatives must focus on multilateral and cross-sectoral cooperation. Often, such cooperation must accommodate high levels of poverty and illiteracy and other substantive barriers to accessing formal health systems.

As we endeavour to combine biomedicine and social medicine to create a trans-disciplinary workforce for the Ebola frontline, we must ensure that our efforts are focused on the people, households and communities at risk. If we are to achieve any global health goals, we must empower the marginalized and voiceless. In the era of globalized supply chains and rapid transportation across very porous borders, it is in our self-interest to recognize our interdependence.

We also need a dose of humility and effective approaches at household, community, societal and global levels. At the household level, we need to promote family-centred interactions and interventions. Cultural practices such as embalming, burial and caregiving are family-based as well as community-based activities.

At community level, we need to re-emphasize the value of partnerships led by trusted community- and faith-based organizations. Even in the best of situations, most of the world’s resource-limited communities tend to be wary of government officials and other outsiders.

At societal level, we need approaches that engage, mobilize and energize non-state, non-political actors while coordinating the ministries involved in health, welfare, finance and education. Grassroots groups with a high reserve of trust can be successfully engaged and motivated to intervene in a manner that is culturally sensitive.

Finally, we need global approaches that will intensify the international response. The global health community should treat the Ebola outbreak as the complex humanitarian emergency that it is.

We admire, commend and thank the tireless and brave frontline workers responding to this tragic outbreak – they are genuine heroes and national treasures. However, without a more effective and robust emergency response – and years of intensive health systems strengthening –there will be many more serious epidemics of Ebola and other infectious diseases. Such epidemics threaten not just the world’s most resource-poor settings but also the entire global community.
